# Squamous cell carcinoma of the eyelid

**DOI:** 10.1093/jjco/hyad127

**Published:** 2023-09-23

**Authors:** Yasuyoshi Sato, Shunji Takahashi, Takashi Toshiyasu, Hideki Tsuji, Nobuhiro Hanai, Akihiro Homma

**Affiliations:** Department of Medical Oncology, The Cancer Institute Hospital of Japanese Foundation for Cancer Research, Tokyo, Japan; Department of Chemotherapy and Cancer Center, The University of Tokyo Hospital, Tokyo, Japan; Department of Medical Oncology, The Cancer Institute Hospital of Japanese Foundation for Cancer Research, Tokyo, Japan; Department of Radiation Oncology, The Cancer Institute Hospital of Japanese Foundation for Cancer Research, Tokyo, Japan; Department of Ophthalmology, The Cancer Institute Hospital of Japanese Foundation for Cancer Research, Tokyo, Japan; Department of Head and Neck Surgery, Aichi Cancer Center Hospital, Nagoya, Japan; Department of Otolaryngology-Head and Neck Surgery, Hokkaido University Graduate School of Medicine, Sapporo, Japan

**Keywords:** eyelid cancer, squamous cell carcinoma, SCC, periocular cancer, cutaneous cancer

## Abstract

Eyelid squamous cell carcinoma is a major type of rare eyelid cancer, together with basal cell carcinoma and sebaceous gland carcinoma. It is a painless disease that progresses slowly and is often detected by the appearance of nodules or plaques. Risk factors include exposure to ultraviolet light, fair skin, radiation and human papillomavirus infection. The standard treatment is surgical removal, and in cases of orbital invasion, orbital content removal is required. If sentinel node biopsy reveals a high risk of lymph node metastasis, adjuvant radiotherapy may be considered. Local chemotherapy, such as imiquimod and 5-fluorouracil, may be used for eyelid squamous cell carcinoma *in situ*. When surgery or radiotherapy is not recommended for distant metastases or locally advanced disease, drug therapy is often according to head and neck squamous cell carcinoma in Japan. The treatment often requires a multidisciplinary team to ensure the preservation of function and cosmetic appearance.

## Teaser

This review is aimed at oncologists and focuses on the classification, epidemiology, examination and diagnosis, treatment and prognosis of rare eyelid cancers, particularly eyelid squamous cell carcinoma.

## Introduction

Eyelid cancer, specifically eyelid squamous cell carcinoma (SCC), is an uncommon type of cancer that may require a team of specialists, such as ocular oncologists, medical oncologists, radiation oncologists, pediatricians, psychologists and other experts, to maintain functionality and cosmetic appearance (1). However, there have been limited comprehensive reports on eyelid SCC from Japan. This review is aimed at oncologists and focuses on eyelid carcinomas, particularly eyelid skin SCC.

## Classification of eyelid cancers

Periocular tumors are classified as eyelid/conjunctival or orbital tumors, each of which has different approaches for diagnosis and treatment (2). The eyelids are divided into anterior and posterior lamellae. The anterior lamella is composed of skin and orbicularis oculi muscle, whereas the posterior lamella is composed of the tarsal plate and palpebral conjunctiva (3). Eyelids possess specialized adnexal structures including (i) meibomian glands in the tarsal plate, (ii) sebaceous glands of Zeis, which open into eyelash follicles and (iii) glands of Moll, which are modified sweat glands that also open into eyelash follicles (4,5). Eyelid cancers can arise from any tissue. Basal cell carcinoma (BCC) arising from skin, sebaceous gland carcinoma (SGC) arising from the meibomian or Zeis glands and SCC arising from skin or ocular conjunctiva are the three most common types. Melanoma, Merkel cell carcinoma and malignant lymphoma are also seen (6).

The TNM Classification of Malignant Tumors, 8th Edition by Union for International Cancer Control (UICC) classifies ‘Carcinoma of Skin of the Eyelid’ separately from ‘Carcinoma of the Skin’ under ‘Skin tumors’ section (Table 1) (7). However, the American Joint Committee on Cancer (AJCC) Cancer Staging Manual, 8th Edition classifies ‘Carcinoma of the Eyelid’ under ‘Ophthalmic Sites’ (Table 2) (8). Both classifications classify conjunctival carcinoma in ‘Ophthalmic Tumors’/‘Ophthalmic Sites’ section. Conjunctival SCC is also an important disease but should be discussed separately; this review only touches on it as necessary.

**Table 1:** Position of eyelid cancer in TNM Classification of Malignant Tumors, 8th Edition by UICC

**Skin tumors**	**Ophthalmic tumors**
**Carcinoma of skin** **Skin carcinoma of the head and neck** **Carcinoma of skin of the eyelid** **Malignant melanoma of skin** Merkel cell carcinoma of skin	Carcinoma of conjunctiva Malignant melanoma of conjunctiva Malignant melanoma of uvea Retinoblastoma Sarcoma of orbit Carcinoma of lacrimal gland

UICC, Union for International Cancer Control.

**Table 2:** TNM staging AJCC UICC 8th edition of eyelid carcinoma

**Category**	**Criteria**		
**Primary tumor (T)**			
TX	Primary tumor cannot be assessed		
T0	No evidence of primary tumor		
Tis	Carcinoma *in situ*		
T1	Tumor ≤10 mm in greatest dimension		
T1a	Tumor does not invade the tarsal plate or eyelid margin		
T1b	Tumor invades the tarsal plate or eyelid margin		
T1c	Tumor involves full thickness of the eyelid		
T2	Tumor >10 mm but ≤20 mm in greatest dimension		
T2a	Tumor does not invade the tarsal plate or eyelid margin		
T2b	Tumor invades the tarsal plate or eyelid margin		
T2c	Tumor involves full thickness of the eyelid		
T3	Tumor >20 mm but ≤30 mm in greatest dimension		
T3a	Tumor does not invade the tarsal plate or eyelid margin		
T3b	Tumor invades the tarsal plate or eyelid margin		
T3c	Tumor involves full thickness of the eyelid		
T4	Any eyelid tumor that invades adjacent ocular, orbital or facial structures		
T4a	Tumor invades ocular or intraorbital structures		
T4b	Tumor invades (or erodes through) the bony walls of the orbit or extends to the paranasal sinuses or invades the lacrimal sac/nasolacrimal duct or brain		
**Regional lymph nodes (N)**			
NX	Regional lymph nodes cannot be assessed		
N0	No evidence of lymph node involvement		
N1	Metastasis in a single ipsilateral regional lymph node, ≤3 cm in greatest dimension		
N1a	Metastasis in a single ipsilateral lymph node based on clinical evaluation or imaging findings		
N1b	Metastasis in a single ipsilateral lymph node based on lymph node biopsy		
N2	Metastasis in a single ipsilateral lymph node, >3 cm in greatest dimension, or in bilateral or contralateral lymph nodes		
N2a	Metastasis documented based on clinical evaluation or imaging findings		
N2b	Metastasis documented based on microscopic findings on lymph node biopsy		
**Distant metastasis (M)**			
M0	No distant metastasis		
M1	Distant metastasis		
Stage	T	N	M
Stage 0	Tis	N0	M0
Stage IA	T1	N0	M0
Stage IB	T2a	N0	M0
Stage IIA	T2b-c, T3	N0	M0
Stage IIB	T4	N0	M0
Stage IIIA	Any T	N1	M0
Stage IIIB	Any T	N2	M0
Stage IV	Any T	Any N	M1

AJCC, American Joint Committee on Cancer.

## Epidemiology of eyelid cancers

Eyelid cancer accounts for 5–10% of all skin cancers (6), and reports from Europe and the USA have indicated that SCC accounts for 5–10% of all skin cancers that occur on the eyelid (9). Eyelid SCC incidence has been reported to range from 0.09 to 2.42 cases per 100 000 people with a high prevalence in males, lower eyelids, especially medial canthus region, fair skinned individuals and countries with high ultraviolet (UV) light exposure (10). Longitudinal studies conducted in the USA and Canada have shown that the age-adjusted incidence of cutaneous SCC has increased by 50–200% over the past several decades (11).

Among eyelid cancers, BCC has relatively low grade histology and is more frequent than SGC and SCC, especially in Europe and the USA (12). Reports from Europe and the USA have indicated that BCC accounts for the majority (83.6–92.2%) of eyelid cancers. SCC is the next most common (7–16.3%), and SGC is less common (0–3%) (10,13–19). Although there are no clear statistics on incidence rates of eyelid cancer in Japan, a review of studies from multiple centers from 1976 to 2004 (*n* = 774) reported 39.5% for BCC, 27.0% for SGC and 21.8% for SCC (20). Subsequent studies from several single centers also revealed higher rates of SGC in Japan than in Western countries, ranging from 16.7 to 43.7% (2,21–23). The percentage of SCCs showed a downward trend from 26.7% in the aforementioned review from 1976 to 1994 to 13.0% during 1995–2004 (20). Later studies from several single centers reported similar results, ranging from 8.5 to 16.7% since 1995 (22). In other Asian countries, SGC frequency is as high as that in Japan with BCC being the most frequent, SGC the second most frequent and SCC the third most frequent (Table 3) (24,25,34–38,26–33).

**Table 3:** Incidence of eyelid cancers by histological type

Distinct	Year	*n*	BCC (%)	SGC (%)	SCC (%)	Reference
Japan	1976–94	498	40.4	23.3	26.7	(20)
	1995–2004	276	38.0	33.7	13.0	
Tochigi (Japan)	1990–2004	24	37.5	37.5	0	(2)
Tokyo (Japan)	Reported 2012	12	33.3	16.7	16.7	(21)
Tokyo (Japan)	1995–2019	412	35.9	43.7	8.5	(22)
Chiba (Japan)	2009–19	NA	27	51	16	(23)
Korea	1976–85	73	35.1	25.2	21.6	(24)
Korea	1993–2016	5655	67.5	10.7	10.6	(25)
China	1955–80	525	47.0	32.7	10.0	(26)
China	2000–18	768	48.7	34.2	12.4	(27)
China	2002–15	292	56.5	34.6	3.8	(28)
China	2010–15	141	59.5	29.0	7.8	(29)
Hong Kong	2000–09	68	43	7	18	(30)
Taiwan	1979–99	1166	65.1	12.6	7.9	(31)
Taiwan	1980–2000	127	62.2	23.6	8.7	(32)
Singapore	1968–95	162	84.0	10.2	3.4	(33)
Thai	2000–4	32	37.5	40.6	6.3	(34)
India	1982–92	178	29.8	32.6	28.0	(35)
India	1957–91	85	38.8	27.1	22.4	(36)
India	1995–2016	536	24	53	18	(37)
India	1996–2016	185	55.7	21.1	10.8	(38)
England	1952–58	104	83.6	0	16.3	(13)
Ireland	2005–15	5457	88.4	<1.0	9.7	(14)
Germany	Reported 1977	252	84.1	0.4	7.9	(15)
Switzerland	1989–2007	5504	86	3	7	(16)
Greece	Reported 2017	129	92.2	0	7.8	(17)
Romania	2000–7	252	39.2	1.1	10.5	(18)
Romania	2016–19	98	87.8	0	12.2	(19)
USA	1976–90	174	90.8	0	8.6	(10)
Australia	1973–82	NA	89.7	9.3	0.5	(97)
Brazil	2014–19	64	61.2	3.0	29.9	(98)
Iran	2000–10	100	83.0	6.0	8.0	(99)
Sudan	1970–75	33	27.2	0	39.3	(100)

BCC, basal cell carcinoma; SGC, sebaceous gland carcinoma; SCC, squamous cell carcinoma.

In Japan, SCC is the second most common form of cutaneous malignancy after BCC. Although statistics on the incidence are unclear, a survey by the Japanese Society for Cutaneous Malignancies reported 2507 cases of cutaneous SCC (28.8% of all cutaneous malignancies) during the 5-year period 1987–1991 with an incidence of ~2.5 cases per 100 000 people annually (39). SCC accounted for 28% of all 29 170 cases of SCC, BCC and melanoma at ~100 institutions from 1987 to 2001. In addition, 8975 cases of actinic keratosis, a precancerous lesion, were included, showing an increasing trend of cutaneous SCC and actinic keratosis (40). Skin cancer incidence is high in highly exposed areas, such as the head, neck and dorsum of the hands, because of increased UV light radiation exposure. More than 80% of nonmalignant melanoma skin cancers occur on the head and neck with 4.6–5.4% occurring on the eyelids. In Australia, adults using sunscreen routinely for 4.5 years have a reduced SCC incidence (2). Childhood sunburn has been reported to increase SCC incidence in Asians (3). In a study examining the distribution of facial skin SCC based on modified facial cosmetic units in Japanese people (*n* = 106), the SCC distribution on the face showed that the cheek (54.2%), frontal region (25.5%) and eyelid (8.5%) were the most common sites of SCC, followed by the lower lip (5.2%), upper lip (3.1%) and nose (3.1%) (41).

## Pathogenesis of eyelid SCCs

Eyelid SCCs arise from skin of the eyelid and ocular conjunctiva. The most common sites are lower and medial eyelids, followed by upper eyelids, but they often involve multiple periocular skin zones. Eyelid SCCs may arise de novo or at the site of precancerous lesions such as actinic keratosis and Bowen’s disease. The major risk factor for tumor development is exposure to UV light radiation. Fair skin, radiation exposure, immunosuppression, a high fat diet, exposure to chemicals (e.g. hydrocarbons and arsenic), smoking and infection by human papillomavirus are also risk factors. The disease is more common in older individuals in their sixties and seventies, and reports from Western countries have indicated that eyelid SCC is 2–3 times more common in men than in women (42,43,44,45). It is important to note that cutaneous SCC is the most common malignancy to develop after solid organ transplantation with a 5-year incidence rate of 30% in lung transplant patients and up to 26% in heart transplant patients (46,47).

Eyelid SCC typically presents as a mass that does not heal spontaneously with various morphologies from small erythematous plaques to papillomatous, nodular or ulcerative lesions that are often painless and progress slowly. As the tumor enlarges, vision may be obstructed, resulting in decreased visual acuity and diplopia may occur due to compression of the eyeball. Early eyelid SCC can present as chronic anterior blepharitis and is easily misdiagnosed (12,6,42). Symptoms of eyelid SCC vary and include a new growth or mass, skin ulcers, eyelash loss, resistant blepharitis or conjunctivitis, tarsal plate thickening, nonspecific eyelid swelling, eyelid discomfort, bleeding or scarring, excessive tearing, a nonhealing stye, wart, or chalazion, violaceous nodules and a pigmented macule with irregular features. Because these clinical features are common to SCC and BCC, they are easily confused (48). Biopsy should be considered when spots on the eyelid change in size, bleed, do not heal, or are a constant source of irritation (43,49).

Cutaneous SCC is highly invasive and may be complicated by local tissue destruction, orbital invasion, perineural invasion, and lymphatic and hematogenous metastases (43). The frequency of microscopic perineural invasion in facial and periorbital SCCs has been reported to be 8–14% (43,50). Eyelid SCC patients with perineural infiltration may present with dysesthesia of trigeminal nerve distribution, ophthalmoplegia, orbital pain and facial paralysis (51). The frequency of regional lymph node metastasis has been reported to be 24%, most commonly in the preauricular, parotid and submandibular glands (42). The frequency of distant lymph node metastasis is reported to be 6.2% (42).

## Examination and diagnosis of eyelid SCCs

On visual examination and slit lamp microscopy, a substantial mass is found on the eyelid. In malignant cases, the mass is often accompanied by malformed protrusions, hair loss and hypervascularization. In most cases, histology can be estimated from clinical findings alone. BCC is often accompanied by pigmentation and ulceration. SGC is yellowish, reflecting fatty deposits, and ridges extend vertically along meibomian glands. SCC is richly vascular with papillary-like structures and is often reddish. Histological diagnosis is usually made by excisional biopsy. SCC appears as nests of squamous cells and is characterized by infiltration by atypical keratinocytes, often with a characteristic keratin pearl. The degree of keratinocyte dysplasia varies from partial thickness keratosis to full thickness intraepidermal carcinoma before invasion of the epidermis (52). The histopathological features of SCC depend on the degree of tumor differentiation. In highly differentiated tumors, cells are polygonal with abundant acidophilic cytoplasm, polychromatic nuclei of varying sizes and staining, dyskeratotic cells and intercellular bridges. In poorly differentiated SCC, there is pleomorphism with undifferentiated cells, an abnormal mitotic picture, little or no keratinization and loss of intercellular bridges (53).

The tumor size, presence or absence of invasion into the angle of the eye and the presence or absence of pagetoid spread are important to determine the extent of resection. Tissue samples should be carefully examined for perineural invasion. Biopsy of the supraorbital nerve may be considered, especially when the trigeminal nerve distribution presents with paresthesias, ophthalmoplegia, orbital pain and facial palsy (54). Palpation should be performed to check for swelling of regional lymph nodes (parotid and cervical), and imaging tests such as CT should be considered when metastasis is suspected.

## Treatment of eyelid cancers

Eyelid cancers can have a devastating effect on a patient’s vision and quality of life, because surgical removal and reconstruction can affect eye functions. Cosmetic considerations are also necessary because the disease and surgical deformity can significantly affect how the patient is viewed by others and themselves, requiring different considerations than other skin cancers of the same histological type (12,52). Treatment of eyelid cancer is considered to save life, sight and the eye, often requiring a multidisciplinary team of ocular oncologists, medical oncologists, radiation oncologists, pediatricians, psychologists and other specialists (1).

## Treatment of locally advanced eyelid SCCs

### Surgery

Surgical resection is the standard treatment for locally advanced eyelid SCCs. The 2020 guidelines for cutaneous malignancies classify patients with eyelid primary SCC as a high-risk group with a recommended safe margin of resection of 6–10 mm or more (55). The 5-year local recurrence rate after surgical resection has been reported to be 2.4–36.9% (42,50). For complete surgical excision using histology to verify tumor-free margins, options include excision with a frozen section control or Mohs surgery (56). ﻿Mohs surgery is an intraoperative pathological examination to verify whether a complete resection has been performed prior to reconstruction. The surgeon performs a tumor resection at what is considered the gross margins of the tumor. The tissue is frozen on a cryostat and sliced horizontally, and sections are prepared from the lowest layer and stained. If tumor cells are found, additional sections are prepared, and the procedure is repeated to remove the minimal amount of tumor tissue. The same procedure can also be used to resect the surrounding margin to minimize the margin. This technique allows a single-stage resection of the tumor with minimal margins (57). The recurrence rate of Mohs surgery has been reported to range from 0 to 3.6% (58). Although it has the potential to be more beneficial than conventional surgical methods, Mohs surgery is not widely used in Japan because of the complexity of the technique, the need for specialized training and the time and labor required for a series of procedures (55). In a German report, the percentage of R1 resection due to failure to achieve R0 resection in Mohs surgery tended to be higher for eyelid SCC [24.3% (17/70)] compared with 16.1% (208/1084) for skin SCC of all sites (59). In addition, when the orbit is involved, removal of the orbital contents is necessary (12,45).

Sentinel node biopsy is considered for eyelid cancers with a risk of regional lymph node metastasis, such as melanoma, SGC and MCC in addition to SCC. It is considered for advanced cancers ≥T2b, because lymph node metastasis has been reported to be present at presentation or during follow-up for eyelid SCCs >18 mm or advanced to AJCC stage T2b (60). In cases with a high risk of lymph node metastasis on sentinel node biopsy, postoperative adjuvant radiotherapy is usually added (12,45).

### Definitive radiotherapy

Definitive radiotherapy is indicated for patients for whom surgery is unsafe or who decline surgery. Treatment is usually administered three to five times a week for 1–2 months. A report of 38 eyelid SCC patients (14 had relapsed disease after prior treatment) who underwent radiation therapy between 1964 and 2010 in a Japanese institution showed that the median radiation dose was 60.0 Gy (range, 45.0–70.4 Gy) and 5-year local and nodal relapse-free, distant metastasis-free and relapse-free rates were 71.8, 77.5, 90.6 and 58.0%, respectively; and the 5-year overall survival was 79.5%; Nine patients complicated Grade 3 or greater late adverse events, and two patients lost their vision (61). Another report of ﻿159 periocular lesions including 18 SCC in 145 patients who underwent radiation therapy between 2009 and 2014 in a single institute in ﻿Switzerland showed a recurrence rate of 11% (62). ﻿For preserving ﻿function and cosmetics, radiation therapy could be a therapeutic option for eyelid SCC.

In clinical practice, lead or tungsten eye shields are often used to protect the cornea, eye, retina and optic nerve, and electron beams are used to achieve both tumor control and functional preservation. Intensity-modulated radiation therapy has also been often used with irradiation methods tailored to the extent of the eyelid conjunctiva and ocular conjunctiva lesions, but there are no published reports.

### Adjuvant radiotherapy

Adjuvant radiotherapy is administered to patients with positive sentinel node biopsies, positive microscopic perineural invasion, positive resection margins or closed margins. The standard radiation dose has not been established, but has been reported to be 26–63 Gy (median: 60 Gy). Known side effects of radiation include local hyperemia, erosion, alopecia, local pain, skin atrophy, pigmentary changes, telangiectasia, dry eye syndrome, conjunctival keratosis, blepharitis, trichiasis, optic neuropathy, retinopathy and keratopathy at the irradiated site (63).

## Treatment of eyelid SCC *in situ*

SCC *in situ* lesions include actinic keratosis and Bowen’s disease. Actinic keratosis is a precancerous lesion or intraepithelial carcinoma of cutaneous SCC that predominantly affects exposed areas of the face, including eyelids and dorsal palms, and 0.025–20% of patients per year progress to SCC when untreated. Bowen’s disease is an intraepidermal lesion of cutaneous SCC, and 3–5% of patients progress to SCC (55). Treatments of these *in situ* cases of eyelid SCC include surgical resection, which is especially recommended for Bowen’s disease (64), local chemotherapy, photodynamic therapy and cryotherapy.

### Local chemotherapy

Topical imiquimod, mitomycin C ophthalmic solution, 5-fluorouracil ophthalmic solution and interferon alpha-2b ophthalmic solution may be used as local chemotherapy for eyelid SCC *in situ* (12,45).

#### Imiquimod

Imiquimod is an agonist of Toll-like receptor 7 that activates production of cytokines (e.g. IFN-α, IL-12 and TNF-α), cellular immunity and apoptosis of tumor cells, and inhibits viral growth.

For actinic keratosis, several clinical trials have reported that topical imiquimod therapy has a lesion resolution rate of 55–85% (65,66). Imiquimod has been approved in Japan for treatment of actinic keratosis limited to the face or bald head area and is recommended in the Japanese guidelines for local treatment of multiple lesions of actinic keratoses. It is also recommended in the Japanese skin malignancy guidelines for local treatment of multiple lesions of actinic keratosis. Studies have demonstrated treatment efficacy of imiquimod for periocular SCC *in situ* (67,68). However, imiquimod has a known risk of conjunctival irritation, but in a retrospective study of topical imiquimod for periocular lesions, the major ophthalmic side effects were temporary conjunctivitis and ocular stinging pain that resolved after completion of imiquimod treatment (69).

For Bowen’s disease, a randomized controlled trial of topical imiquimod reported a 73% resolution rate of lesions after 12 weeks of treatment (70). Therapeutic effects on periocular lesions have also been reported (71), but as of April 2023, it is not covered by national insurance in Japan for Bowen’s disease.

There is also a report of an elderly patient with locally advanced eyelid SCC who achieved CR after local chemotherapy with imiquimod alone (72), and this may be an option for locally advanced cases for which surgery is not indicated.

#### 5-Fluorouracil

Topical 5-fluorouracil is recommended in the Japanese guidelines for cutaneous malignancies for multiple thin lesions of actinic keratosis (55). The reported resolution rate of lesions ranges from 26 to 96%, and the incidence of local adverse reactions varies somewhat from 25 to 77%. The most common side effects include skin irritation and hyperemia (73,74,75,65,76).

#### Mitomycin C

Topical mitomycin C is not mentioned in the Japanese guidelines for cutaneous malignancies. In a retrospective report of patients with ocular surface SCC who received topical 5-fluorouracil (*n* = 89) or mitomycin C (*n* = 64) for residual disease after surgical excision, there was one recurrence in the 5-fluorouracil group and zero in the mitomycin C group. Adverse effects occurred in 69% and 41% of patients, respectively, but no sight-threatening complications were noted (77).

### Cryotherapy

Cryotherapy destroys the tissue structure of SCC using liquid nitrogen at very low temperatures. Cryotherapy with liquid nitrogen is recommended as a simple and effective treatment for actinic keratosis in the Japanese guidelines for cutaneous malignancies (55). The complete resolution rate is 68–86%, and the local side effect rate is 35–43% (73,74,75,78) (79,80). The main side effects include localized pain, swelling, pigment changes, hair loss in hairy areas, blister formation and scarring (81,73,78,79) (80).

### Photodynamic therapy

Photodynamic therapy uses photosensitizers, light and oxygen to induce targeted cell death by apoptosis in cancer and abnormal tissues. It is recommended in the Japanese guidelines for cutaneous malignancies for the treatment of extensive multiple lesions of actinic keratosis (55), but it is not covered by national insurance in Japan. The local lesion resolution rate is 68–93%, and the incidence of local adverse reactions is 26–100%, including minor reactions such as local erythema, although the incidence of adverse reactions is somewhat high (73,79,80,66).

## Treatment of metastatic eyelid SCC

No randomized controlled trials of chemotherapy have been conducted for unresectable advanced or metastatic cutaneous SCC including eyelid SCC, and there is no standard of care. Eyelid SCC is often treated by chemotherapy similar to head and neck SCC.

For chemotherapy of cutaneous SCC, cisplatin is considered the main drug, and it is also used for head and neck SCC (65). In a report of 14 patients treated with fluorouracil (5-FU) and cisplatin, a response was seen in 11 patients (82). The degree of epidermal growth factor receptor (EGFR) overexpression is associated with the aggressiveness of cutaneous SCC and has been reported to be an independent predictor of metastasis in an analysis of 54 cases of head and neck SCC (83). Cetuximab, an anti-EGFR antibody, has been shown to be effective for head and neck SCC in several clinical trials (84,85,86). It is also used in definitive chemoradiotherapy for locally advanced head and neck SCC and in palliative chemotherapy for unresectable advanced or recurrent head and neck SCC. There is no evidence for the efficacy of anti-EGFR antibody drugs in eyelid SCC patients. However, for overall cutaneous SCC, a phase II trial of single-agent cetuximab in 36 cutaneous SCC patients, including five primary head and neck SCC patients, showed disease control in 25 patients (69%) (87). A retrospective study of five conjunctival SCC cases showed moderate to strong expression of EGFR in both the invasive and *in situ* components in all cases (83). In addition, there was a report of response to the EGFR tyrosine kinase inhibitor erlotinib in two elderly patients with skin SCC with orbital involvement (88).

In head and neck SCC, the anti-programmed death receptor-1 (PD-1) antibody nivolumab has shown efficacy in platinum-resistant patients (89,90), and the anti-PD-1 antibody pembrolizumab has shown efficacy in platinum-sensitive patients (91,92) and palliative chemotherapy for unresectable advanced or recurrent head and neck SCC. Immune checkpoint inhibitors have been used as single agents or in combination with chemotherapy, but there is no evidence for the efficacy of immune checkpoint inhibitors in eyelid cancer patients. A phase 2 trial of cemiplimab, an anti-PD-1 antibody, demonstrated a response in 28 (47%) of 59 cutaneous SCC patients, including 38 head and neck primary cases, and 16 (57%) of the 28 responders had a sustained response for >6 months (93). The US FDA approved cemiplimab for cutaneous SCC in September 2018. In a retrospective study of 31 patients with conjunctival SCC, including conjunctival and primary eyelid SCC, 14 patients (47%) showed PD-L1 expression (≥1% of tumor cells) and the density of CD8-positive T cells was higher in advanced stage tumors and that of HPV-positive T cells was higher in primary SCC tumors and HPV-positive tumors (94).

On the basis of this evidence for head and neck SCC and cutaneous SCC, and considering drug accessibility because of the insurance system in Japan, it appears reasonable to use chemotherapy similar to that for head and neck SCC for unresectable and advanced eyelid SCC.

## Biomarkers of eyelid SCC

Approximately one-third of 85 patients with periocular SCC showed p63 expression. The p63 expression was associated with poor differentiation (*P* = 0.029), a high risk of perineural invasion (*P* = 0.042, OR = 4.61) and metastasis (*P* = 0.009, HR = 3.99). Expression of p63 (*P* = 0.012, HR = 7.80), co-expression of p63 and Ki67 (*P* = 0.007, HR = 9.21) and distant metastasis (*P* = 0.001, HR = 11.23) were associated with disease-specific death (95).

## Prognosis and surveillance of eyelid SCC

Patients with eyelid malignancies require long-term follow-up even when resection margins are negative. The recommended frequency and duration of follow-up is 5 years postoperatively for SCC. The local recurrence rate at 5 years after surgical resection has been reported to be 2.4–36.9% (12,45) In all cases of SCC, they could recur, and patients should be encouraged to participate in lifelong follow-up.

There are no prognostic reports stratified by stage. In a retrospective report from Germany of 117 patients with eyelid SCC, including 12 distant metastases, between January 2009 and March 2020, 88 patients (75.2%) underwent radical resection, and the remaining patients underwent surgery plus individualized adjuvant therapy. Disease-specific survival was 95.7% at 2 years and 94.9% at 5 years. Six patients (5.1%) died from eyelid SCC with nodal metastasis and a high T-category being negative prognostic factors. Recurrence was high in patients with multiple lesions, and mortality was associated with the tumor location on the medial side of the upper eyelid and more lymph node metastases (96).

Perineural invasions are a sign of a poor prognosis. Microscopic perineural invasion is reported to be 8–14% (12,45). Therefore, these patients need to be followed more closely (43).

The frequency of visits depends on many factors, including the cancer stage at the time of the initial visit. At each visit, the surgical site should be examined clinically for signs of recurrence. Patients who have undergone orbital resection for advanced cancer of the eyelid or periocular region should have imaging of the orbit performed during the follow-up period. Most nodal metastases occur within 2–3 years after cancer treatment (12).

## Conclusion

Eyelid SCC is a rare cancer that requires a multidisciplinary team of specialists, and oncologist should organize their findings. The standard treatment for locally advanced cases is surgery (with the addition of postoperative radiation for sentinel node-positive cases), but there is a lack of evidence regarding the role of definitive radiation therapy, local treatment of *in situ* disease and drug therapy for distant metastases and recurrent disease. Constructing evidence for eyelid SCC is warranted.

## References

[1] Maheshwari A, Finger PT. Cancers of the eye. Cancer Metastasis Rev 2018;37:677–90. 30203109 10.1007/s10555-018-9762-9

[2] Obata H, Aoki Y, Kubota S, Kanai N, Tsuru T. Incidence of benign and malignant lesions of eyelid and conjunctival tumors. Nihon Ganka Gakkai Zasshi 2005;109:573–9. 16218435

[3] Blumenthal SR, Swick M, Bayan CA, Ramanathan D, Maher I. Complex eyelid reconstruction: a practical guide for the Mohs surgeon. Dermatologic Surg 2022;48:916–23. 10.1097/DSS.0000000000003526 36054043

[4] Kels BD, Grzybowski A, Grant-Kels JM. Human ocular anatomy. Clin Dermatol 2015;33:140–6. 25704934 10.1016/j.clindermatol.2014.10.006

[5] Monheit G, Hrynewycz K. Mohs surgery for periocular tumors. Dermatol Surg 2019;45:S70–8. 31764293 10.1097/DSS.0000000000002254

[6] Sullivan TJ, Boulton JE, Whitehead KJ. Intraepidermal carcinoma of the eyelid. Clin Exp Ophthalmol 2002;30:23–7. 11885790 10.1046/j.1442-9071.2002.00486.x

[7] Brierley JD, Gospodarowicz MK, Wittekind C. TNM Classification of Malignant Tumours, 8th edn. New Jersey: Wiley-Blackwell, 2016.

[8] Amin MB, Edge SB, Greene FL, et al., editor. AJCC Cancer Staging Manual, 8th edn. Cham: Springer, 2017.

[9] Cook BE, Bartley GB. Treatment options and future prospects for the management of eyelid malignancies an evidence-based update. Ophthalmology 2001;108:2088–98. 11713084 10.1016/s0161-6420(01)00796-5

[10] Cook BE, Bartley GB. Epidemiologic characteristics and clinical course of patients with malignant eyelid tumors in an incidence cohort in Olmsted County, Minnesota. Ophthalmology 1999;106:746–50. 10201597 10.1016/S0161-6420(99)90161-6

[11] Gallagher RP, Ma B, McLean DI, et al. Trends in basal cell carcinoma, squamous cell carcinoma, and melanoma of the skin from 1973 through 1987. J Am Acad Dermatol 1990;23:413–21. 2212139 10.1016/0190-9622(90)70234-9

[12] Yin VT, Merritt HA, Sniegowski M, Esmaeli B. Eyelid and ocular surface carcinoma: diagnosis and management. Clin Dermatol 2015;33:159–69. 25704936 10.1016/j.clindermatol.2014.10.008

[13] Halnan KE, Britten MJ. Late functional and cosmetic results of treatment of eyelid tumours. Br J Ophthalmol 1968;52:43–53. 5710508 10.1136/bjo.52.1.43 PMC506521

[14] Quigley C, Deady S, Hughes E, McElnea E, Zgaga L, Chetty S. National incidence of eyelid cancer in Ireland (2005–2015). Eye 2019;33:1534–9. 30976073 10.1038/s41433-019-0437-8 PMC7002498

[15] Lentrodt J . Principles of the surgical therapy of eyelid tumours. J Maxillofac Surg 1977;5:93–107. 267708 10.1016/s0301-0503(77)80084-2

[16] Deprez M, Uffer S. Clinicopathological features of eyelid skin tumors. A retrospective study of 5504 cases and review of literature. Am J Dermatopathol 2009;31:256–62. 19384066 10.1097/DAD.0b013e3181961861

[17] Tzoutzos K, Batistatou A, Kitsos G, Liasko R, Stefanou D. Retrospective clinicopathological study of 129 cancerous and 18 precancerous lesions of the eyelids in North-Western Greece. Int Ophthalmol 2017;37:203–8. 27209420 10.1007/s10792-016-0258-8

[18] Coroi MC, Roşca E, Muţiu G, Coroi T, Bonta M. Eyelid tumors: histopathological and clinical study performed in county hospital of Oradea between 2000-2007. Rom J Morphol Embryol 2010;51:111–5. 20191129

[19] Mercut IM, LCl I, Tanasie CA, et al. Analysis of tumour related data and clinical features of eyelid carcinomas. Curr Heal Sci J 2020;46:222–9. 10.12865/CHSJ.46.03.02 PMC7716762 33304622

[20] Takamura H, Yamashita H. Clinicopathological analysis of malignant eyelid tumor cases at Yamagata University Hospital: statistical comparison of tumor incidence in Japan and in other countries. Jpn J Ophthalmol 2005;49:349–54. 16187033 10.1007/s10384-005-0229-5

[21] Naoki, Mamada HT . Incidence of benign and malignant eyelid tumors in Japan. Int J Ophthalmic Pathol 2012;01:2. 10.4172/2324-8599.1000102.

[22] Goto H, Yamakawa N, Komatsu H, et al. Epidemiological characteristics of malignant eyelid tumors at a referral hospital in Japan. Jpn J Ophthalmol 2022;66:343–9. 35670924 10.1007/s10384-022-00926-z

[23] Shimizu N, Oshitari T, Yotsukura J, Yokouchi H, Baba T, Yamamoto S. Ten-year epidemiological study of ocular and orbital tumors in Chiba University Hospital. BMC Ophthalmol 2021;21:1–8. 34556080 10.1186/s12886-021-02108-w PMC8459513

[24] Roh KK, Lee JH, Youn DH. Clinical analysis of tumors of the eye and its adnexa. Korean J Ophthalmol 1988;2:27–31. 3071636 10.3341/kjo.1988.2.1.27

[25] Jung SK, Lim J, Yang SW, Jee D, Won YJ. Nationwide trends in the incidence and survival of eyelid skin cancers in Korea. Ophthalmic Epidemiol 2020;27:438–48. 32486892 10.1080/09286586.2020.1767152

[26] Ni C, Searl SS, Kuo PK, Chu FR, Chong CS, Albert DM. Sebaceous cell carcinomas of the ocular adnexa. Int Ophthalmol Clin 1982;22:23–61. 7061198 10.1097/00004397-198202210-00006

[27] Wang L, Shan Y, Dai X, et al. Clinicopathological analysis of 5146 eyelid tumours and tumour-like lesions in an eye centre in South China, 2000-2018: a retrospective cohort study. BMJ Open 2021;11:e041854–8. 10.1136/bmjopen-2020-041854 PMC7839916 33500284

[28] Yu SS, Zhao Y, Zhao H, Lin JY, Tang X. A retrospective study of 2228 cases with eyelid tumors. Int J Ophthalmol 2018;11:1835–41. 30450316 10.18240/ijo.2018.11.16 PMC6232338

[29] Han Y, Kong M, Luo Y, et al. Clinicopathological features of patients with wide local excision of eyelid malignant neoplasms: a more than five years retrospective study from China. BMC Ophthalmol 2022;22:1–10. 36376823 10.1186/s12886-022-02645-y PMC9664722

[30] Ho M, Liu DTL, Chong KKL, et al. Eyelid tumours and pseudotumours in Hong Kong: a ten-year experience. Hong Kong Med J 2013;19:150–5. 23535675

[31] Lin HY, Cheng CY, Hsu WM, Kao WHL, Chou P. Incidence of eyelid cancers in Taiwan. A 21-year review. Ophthalmology 2006;113:2101–7. 16962174 10.1016/j.ophtha.2006.06.001

[32] Wang JK, Liao SL, Jou JR, et al. Malignant eyelid tumours in Taiwan. Eye 2003;17:216–20. 12640409 10.1038/sj.eye.6700231

[33] Lee SB, Saw SM, Hong KGA, et al. Incidence of eyelid cancers in Singapore from 1968 to 1995. Br J Ophthalmol 1999;83:595–7. 10216061 10.1136/bjo.83.5.595 PMC1723038

[34] Pornpanich K, Chindasub P. Eyelid tumors in Siriraj hospital from 2000-2004. J Med Assoc Thai 2005;88 Suppl 9:S11–4. 16681045

[35] Sihota R . Malignant eyelid tumors in an Indian population. Arch Ophthalmol 1996;114:108. 8540844 10.1001/archopht.1996.01100130104031

[36] Abdi U, Tyagi N, Maheshwari V, et al. Tumours of eyelid: a clinicopathologic study. J Indian Med Assoc 1996;94:405–9 416, 418. 9141864

[37] Kaliki S, Bothra N, Bejjanki KM, et al. Malignant eyelid tumors in India: a study of 536 Asian Indian patients. Ocul Oncol Pathol 2019;5:210–9. 31049330 10.1159/000491549 PMC6489076

[38] Banerjee P, Koka K, Alam M, et al. The spectrum and clinicopathological correlation of eyelid lesions: twenty years’ experience at a tertiary eye care center in South India. Indian J Ophthalmol 2022;70:43. 34937206 10.4103/ijo.IJO_428_21 PMC8917552

[39] Ishihara K, Ikeda S, Mori S. Hifuakuseisyuyou no shindan to chiryoushishin narabini zenkokuannke-to no syuukei (in Japanese). Skin Cancer 1994;9:99–105.

[40] Ishihara K . Past statistics and prognostic factors for malignant skin tumors. Skin Cancer 2007;22:209–16.

[41] Kato H, Oda T, Watanabe S, Morita A. Facial distribution of squamous cell carcinoma in Japanese. Exp Dermatol 2019;28:72–4. 10.1111/exd.13830 30698883

[42] Faustina M, Diba R, Ahmadi MA, Gutstein BF, Esmaeli B. Patterns of regional and distant metastasis in patients with eyelid and periocular squamous cell carcinoma. Ophthalmology 2004;111:1930–2. 15465559 10.1016/j.ophtha.2004.02.009

[43] Donaldson MJ, Sullivan TJ, Whitehead KJ, Williamson RM. Squamous cell carcinoma of the eyelids. Br J Ophthalmol 2002;86:1161–5. 12234899 10.1136/bjo.86.10.1161 PMC1771324

[44] Neale RE, Weissenborn S, Abeni D, et al. Human papillomavirus load in eyebrow hair follicles and risk of cutaneous squamous cell carcinoma. Cancer Epidemiol Biomarkers Prev 2013;22:719–27. 23396961 10.1158/1055-9965.EPI-12-0917-T

[45] Silverman N, Shinder R. What’s new in eyelid tumors. Asia-Pacific J Ophthalmol 2017;6:143–52. 10.22608/APO.201701 28399340

[46] Singer JP, Boker A, Metchnikoff C, et al. High cumulative dose exposure to voriconazole is associated with cutaneous squamous cell carcinoma in lung transplant recipients. J Hear Lung Transplant 2012;31:694–9. 10.1016/j.healun.2012.02.033 PMC3371090 22484291

[47] Alam M, Brown RN, Silber DH, et al. Increased incidence and mortality associated with skin cancers after cardiac transplant. Am J Transplant 2011;11:1488–97. 21718441 10.1111/j.1600-6143.2011.03598.x

[48] Kersten RC, Ewing-Chow D, Kulwin DR, Gallon M. Accuracy of clinical diagnosis of cutaneous eyelid lesions. Ophthalmology 1997;104:479–84. 9082276 10.1016/s0161-6420(97)30288-7

[49] Harvey DT, Taylor RS, Itani KM, Loewinger RJ. Mohs micrographic surgery of the eyelid: an overview of anatomy, pathophysiology, and reconstruction options. Dermatologic Surg 2013;39:673–97. 10.1111/dsu.12084 23279119

[50] Goepfert H, Dichtel WJ, Medina JE, Lindberg RD, Luna MD. Perineural invasion in squamous cell skin carcinoma of the head and neck. Am J Surg 1984;148:542–7. 6486325 10.1016/0002-9610(84)90385-4

[51] Bowyer JD, Sullivan TJ, Whitehead KJ, Kelly LE, Allison RW. The management of perineural spread of squamous cell carcinoma to the ocular adnexae. Ophthal Plast Reconstr Surg 2003;19:275–81. 10.1097/01.IOP.0000075795.19917.B5 12878875

[52] Moran JM, Phelps PO. Periocular skin cancer: diagnosis and management. Dis Mon 2020;66:101046. 32600650 10.1016/j.disamonth.2020.101046

[53] Pe’er J . Pathology of eyelid tumors. Indian J Ophthalmol 2016;64:177–90. 27146927 10.4103/0301-4738.181752 PMC4869455

[54] Esmaeli B, Ahmadi MA, Gillenwater AM, Faustina MM, Amato M. The role of supraorbital nerve biopsy in cutaneous malignancies of the periocular region. Ophthal Plast Reconstr Surg 2003;19:282–6. 10.1097/01.IOP.0000075018.83519.02 12878876

[55] Ansai S, Umebayashi Y, Katsumata N, et al. Japanese dermatological association guidelines: outlines of guidelines for cutaneous squamous cell carcinoma 2020. Jpn J Dermatol 2020;130:2501–33. 10.1111/1346-8138.15889 33963604

[56] Mehta VJ, Ling J, Sobel RK. Review of targeted therapies for periocular tumors. Int Ophthalmol Clin 2017;57:153–68. 27898621 10.1097/IIO.0000000000000149

[57] Chen ELA, Srivastava D, Nijhawan RI. Mohs micrographic surgery: development, technique, and applications in cutaneous malignancies. Semin Plast Surg 2018;32:060–8. 10.1055/s-0038-1642057 PMC5951690 29765269

[58] Mendoza PR, Grossniklaus HE. Sentinel lymph node biopsy for eyelid and conjunctival tumors: what is the evidence? Int Ophthalmol Clin 2015;55:123–36. 10.1097/IIO.0000000000000051 PMC4548266 25436498

[59] Warnig C, Wollina U. Cutaneous squamous cell carcinoma of head and neck region: a single center analysis of 1296 tumors with clinical characteristics, comorbidities, treatment, and sun-protection behavior. Dermatol Ther 2021;34:1–7. 10.1111/dth.14992 34009659

[60] Nasser QJ, Roth KG, Warneke CL, Yin VT, el Sawy T, Esmaeli B. Impact of AJCC “T” designation on risk of regional lymph node metastasis in patients with squamous carcinoma of the eyelid. Br J Ophthalmol 2014;98:498–501. 24429279 10.1136/bjophthalmol-2013-304434

[61] Inaba K, Ito Y, Suzuki S, et al. Results of radical radiotherapy for squamous cell carcinoma of the eyelid. J Radiat Res 2013;54:1131–7. 23750022 10.1093/jrr/rrt069 PMC3823788

[62] Lazarevic D, Ramelyte E, Dummer R, Imhof L. Radiotherapy in periocular cutaneous malignancies: a retrospective study. Dermatology 2019;235:234–9. 30939473 10.1159/000496539

[63] Hsu A, Frank SJ, Ballo MT, et al. Postoperative adjuvant external-beam radiation therapy for cancers of the eyelid and conjunctiva. Ophthal Plast Reconstr Surg 2008;24:444–9. 10.1097/IOP.0b013e31818be098 19033839

[64] Bernardini FP . Management of malignant and benign eyelid lesions. Curr Opin Ophthalmol 2006;17:480–4. 16932064 10.1097/01.icu.0000243022.20499.90

[65] Krawtchenko N, Roewert-Huber J, Ulrich M, Mann I, Sterry W, Stockfleth E. A randomised study of topical 5% imiquimod vs. topical 5-fluorouracil vs. cryosurgery in immunocompetent patients with actinic keratoses: a comparison of clinical and histological outcomes including 1-year follow-up. Br J Dermatol 2007;157:34–40. 18067630 10.1111/j.1365-2133.2007.08271.x

[66] Sotiriou E, Apalla Z, Maliamani F, Zaparas N, Panagiotidou D, Ioannides D. Intraindividual, right-left comparison of topical 5-aminolevulinic acid photodynamic therapy vs. 5% imiquimod cream for actinic keratoses on the upper extremities. J Eur Acad Dermatology Venereol 2009;23:1061–5. 10.1111/j.1468-3083.2009.03259.x 19470041

[67] Toso F, Tronconi MC, Cortese A, et al. Complete response of locally advanced cutaneous squamous cell carcinoma of the eyelid to topical imiquimod 3.75%. Dermatol Ther 2022;35:3–5. 10.1111/dth.15800 PMC9786355 36066074

[68] Ross AH, Kennedy CTC, Collins C, Harrad RA. The use of imiquimod in the treatment of periocular tumours. Orbit 2010;29:83–7. 20394545 10.3109/01676830903294909

[69] Cannon PS, O’Donnell B, Huilgol SC, et al. The ophthalmic side-effects of imiquimod therapy in the management of periocular skin lesions. Br J Ophthalmol 2011;95:1682–5. 20702431 10.1136/bjo.2009.178202

[70] Patel GK, Goodwin R, Chawla M, et al. Imiquimod 5% cream monotherapy for cutaneous squamous cell carcinoma in situ (Bowen’s disease): a randomized, double-blind, placebo-controlled trial. J Am Acad Dermatol 2006;54:1025–32. 16713457 10.1016/j.jaad.2006.01.055

[71] Brannan PA, Anderson HK, Kersten RC, Kulwin DR. Bowen disease of the eyelid successfully treated with imiquimod. Ophthalmic Plast Reconstr Surg 2005;21:321–2. 16052154 10.1097/01.iop.0000170421.07098.61

[72] Singh M, Singh H, Kakkar N, Zadeng Z, Gupta P. Treatment of squamous cell carcinoma of the eyelid with imiquimod 5% cream. Can J Ophthalmol 2019;54:e24–7. 30851791 10.1016/j.jcjo.2018.03.010

[73] Morton C, Horn M, Leman J, et al. Comparison of topical methyl aminolevulinate photodynamic therapy with cryotherapy or fluorouracil for treatment of squamous cell carcinoma in situ: results of a multicenter randomized trial. Arch Dermatol 2006;142:729–35. 16785375 10.1001/archderm.142.6.729

[74] Walker JL, Siegel JA, Sachar M, et al. 5-fluorouracil for actinic keratosis treatment and chemoprevention: a randomized controlled trial. J Invest Dermatol 2017;137:1367–70. 28532759 10.1016/j.jid.2016.12.029

[75] Cunningham TJ, Tabacchi M, Eliane JP, et al. Randomized trial of calcipotriol combined with 5-fluorouracil for skin cancer precursor immunotherapy. J Clin Invest 2017;127:106–16. 27869649 10.1172/JCI89820 PMC5199703

[76] Hantash BM, Stewart DB, Cooper ZA, Rehmus WE, Koch RJ, Swetter SM. Facial resurfacing for nonmelanoma skin cancer prophylaxis. Arch Dermatol 2006;142:976–82. 16924046 10.1001/archderm.142.8.976

[77] Bahrami B, Greenwell T, Muecke JS. Long-term outcomes after adjunctive topical 5-flurouracil or mitomycin C for the treatment of surgically excised, localized ocular surface squamous neoplasia. Clin Exp Ophthalmol 2014;42:317–22. 23927413 10.1111/ceo.12184

[78] Freeman M, Vinciullo C, Francis D, et al. A comparison of photodynamic therapy using topical methyl aminolevulinate (Metvix®) with single cycle cryotherapy in patients with actinic keratosis: a prospective, randomized study. J Dermatolog Treat 2003;14:99–106. 12775317 10.1080/09546630310012118

[79] Szeimies RM, Karrer S, Radakovic-Fijan S, et al. Photodynamic therapy using topical methyl 5-aminolevulinate compared with cryotherapy for actinic keratosis: a prospective, randomized study. J Am Acad Dermatol 2002;47:258–62. 12140473

[80] Zane C, Facchinetti E, Rossi MT, Specchia C, Ortel B, Calzavara-Pinton P. Cryotherapy is preferable to ablative CO2 laser for the treatment of isolated actinic keratoses of the face and scalp: a randomized clinical trial. Br J Dermatol 2014;170:1114–21. 24472087 10.1111/bjd.12847

[81] Lisman RD, Jakobiec FA, Small P. Sebaceous carcinoma of the eyelids. Ophthalmology 1989;96:1021–6. 2475841 10.1016/s0161-6420(89)32798-9

[82] Sadek H, Azli N, Wendling JL, et al. Treatment of advanced squamous cell carcinoma of the skin with cisplatin, 5-fluorouracil, and bleomycin. Cancer 1990;66:1692–6. 1698529 10.1002/1097-0142(19901015)66:8<1692::aid-cncr2820660807>3.0.co;2-y

[83] Shepler TR, Prieto VG, Diba R, Neuhaus RW, Shore JW, Esmaeli B. Expression of the epidermal growth factor receptor in conjunctival squamous cell carcinoma. Ophthal Plast Reconstr Surg 2006;22:113–5. 10.1097/01.iop.0000202609.92772.c3 16550055

[84] Vermorken JB, Mesia R, Rivera F, et al. Platinum-based chemotherapy plus cetuximab in head and neck cancer. N Engl J Med 2008;359:1116–27. 18784101 10.1056/NEJMoa0802656

[85] Hitt R, Irigoyen A, Cortes-Funes H, Grau JJ, García-Sáenz JA, Cruz-Hernandez JJ. Phase II study of the combination of cetuximab and weekly paclitaxel in the first-line treatment of patients with recurrent and/or metastatic squamous cell carcinoma of head and neck. Ann Oncol 2012;23:1016–22. 21865152 10.1093/annonc/mdr367

[86] Bonner JA, Harari PM, Giralt J, et al. Radiotherapy plus cetuximab for squamous-cell carcinoma of the head and neck. 2006;567–78. https://doi.org/101056/NEJMoa053422. 10.1056/NEJMoa053422 16467544

[87] Maubec E, Petrow P, Scheer-Senyarich I, et al. Phase II study of cetuximab as first-line single-drug therapy in patients with unresectable squamous cell carcinoma of the skin. J Clin Oncol 2011;29:3419–26. 21810686 10.1200/JCO.2010.34.1735

[88] El-Sawy T, Sabichi AL, Myers JN, et al. Epidermal growth factor receptor inhibitors for treatment of orbital squamous cell carcinoma. Arch Ophthalmol 2012;130:1608–11. 23229707 10.1001/archophthalmol.2012.2515

[89] Ferris RL, Blumenschein G, Fayette J, et al. Nivolumab for recurrent squamous-cell carcinoma of the head and neck. N Engl J Med 2016;375:1856–67. 27718784 10.1056/NEJMoa1602252 PMC5564292

[90] Ferris RL, Blumenschein G, Fayette J, et al. Nivolumab vs investigator’s choice in recurrent or metastatic squamous cell carcinoma of the head and neck: 2-year long-term survival update of CheckMate 141 with analyses by tumor PD-L1 expression. Oral Oncol 2018;81:45–51. 29884413 10.1016/j.oraloncology.2018.04.008 PMC6563923

[91] Burtness B, Harrington KJ, Greil R, et al. Pembrolizumab alone or with chemotherapy versus cetuximab with chemotherapy for recurrent or metastatic squamous cell carcinoma of the head and neck (KEYNOTE-048): a randomised, open-label, phase 3 study. Lancet 2019;394:1915–28. 31679945 10.1016/S0140-6736(19)32591-7

[92] Harrington KJ, Burtness B, Greil R, et al. Pembrolizumab with or without chemotherapy in recurrent or metastatic head and neck squamous cell carcinoma: updated results of the phase III KEYNOTE-048 study. J Clin Oncol 2023;41:790–802. 36219809 10.1200/JCO.21.02508 PMC9902012

[93] Migden MR, Rischin D, Schmults CD, et al. PD-1 blockade with Cemiplimab in advanced cutaneous squamous-cell carcinoma. N Engl J Med 2018;379:341–51. 29863979 10.1056/NEJMoa1805131

[94] Nagarajan P, El-Hadad C, Gruschkus SK, et al. PD-L1/PD1 expression, composition of tumor-associated immune infiltrate, and HPV status in conjunctival squamous cell carcinoma. Investig Opthalmology Vis Sci 2019;60:2388–98. 10.1167/iovs.19-26894 PMC6890426 31141610

[95] Luo Y, Chen H, Ge S, et al. Clinical and pathological risk factors for worse stage and mortality of eyelid and periocular squamous cell carcinoma. Br J Ophthalmol 2022;106:1338–43. 33879470 10.1136/bjophthalmol-2020-317546

[96] Klingenstein A, Samel C, Messmer EM, Garip-Kuebler A, Priglinger SG, Hintschich CR. Epidemiological characteristics and clinical course of eyelid squamous cell carcinoma patients from a large tertiary centre between 2009 and 2020. Br J Ophthalmol 2022;106:1057–62. 33712477 10.1136/bjophthalmol-2020-317969 PMC9340003

[97] Francis IC, Benecke PS, Kappagoda MB. A ten-year hospital survey of eyelid cancer. Aust J Ophthalmol 1984;12:121–7. 6487181

[98] Dias MR, Osaki MH, Ferreira CAA, et al. Epidemiological Profile and Clinical Stage at Presentation of Eyelid Malignancies in a Multi-Ethnic Country. J Craniofac Surg 2021;32:E642–5. 33852518 10.1097/SCS.0000000000007649

[99] Tavakoli M, Zavareh R, Aletaha M, et al. Eyelid masses: A 10-year survey from a tertiary eye hospital in Tehran. Middle East Afr J Ophthalmol 2013;20:187. 10.4103/0974-9233.114788 PMC3757624 24014978

[100] Malik MOA, El Sheikh EH. Tumors of the eye and adnexa in the Sudan. Cancer 1979;44:293–303. 10.1002/1097-0142(197907)44:1<293::aid-cncr2820440150>3.0.co;2-r 455254

